# Commentary: Gain in Body Fat Is Associated with Increased Striatal Response to Palatable Food Cues, whereas Body Fat Stability Is Associated with Decreased Striatal Response

**DOI:** 10.3389/fnhum.2017.00065

**Published:** 2017-02-14

**Authors:** Silvia Cerolini, Mariella Pazzaglia, Caterina Lombardo

**Affiliations:** ^1^Department of Psychology, La Sapienza University of RomeRome, Italy; ^2^IRCCS Santa Lucia FoundationRome, Italy

**Keywords:** reward, food-cues, adolescence, striatal response, weight gain

## Perspectives on neural vulnerability to weight gain during adolescence

Obesity has become a global public health challenge in the last few years (Swinburn et al., 2011). Body fat stability is particularly important for preventing future weight gain, and its influence is felt as early as adolescence. Behavioral studies have suggested that palatability and access to a variety of foods may affect appetite, food intake, and weight gain (Johnson and Wardle, 2014). Indeed, it has been demonstrated that food-cue reactivity and cue-induced cravings predict eating habits and weight gain, both systematically and prospectively (Boswell and Kober, 2016).

From a neural perspective, some regions of the brain involved in reward (the ventral striatum and orbitofrontal cortex) and attention (anterior cingulate cortex and precuneus) undergo pronounced changes in response to high-calorie food cues (Small et al., 2001; Berridge et al., 2010; Ziauddeen et al., 2015; Stice and Yokum, 2016a). Although the relationship between the neural circuits activated during food intake or food-cue reactivity and weight gain has been studied in depth, there are few longitudinal studies on the development of food hyper-responsivity during adolescence and neural vulnerability factors (Holsen et al., 2005; Killgore and Yurgelun-Todd, 2005; Yokum et al., 2014).

An article by Stice and Yokum (2016b) published in the *Journal of Neuroscience* focuses on the link between weight gain (body fat percentage) and the responsivity of neural regions involved in food reward. A repeated-measures fMRI protocol was applied in this longitudinal study of adolescents to assess changes in the blood-oxygen-level dependent (BOLD) signal response within the regions of the brain involved in reward and attention to palatable food cues when the participants gained weight, lost weight, or maintained a stable weight over a two- to three-year follow-up period. As a control procedure, the researchers studied neural responses to the receipt and potential receipt of monetary rewards among the same group of adolescents. The authors found increased activation of the putamen, mid-insula, and Rolandic operculum in response to palatable food images among participants who gained body fat compared to those who remained stable or lost weight after a 2 or 3-year follow-up period.

This finding is in line with other studies that found the activation of these regions to be increased in response to palatable food cues among people with high body mass indices (Stoeckel et al., 2008; Martin et al., 2010; Stice et al., 2010). These studies also align with incentive sensitization theory, according to which elevated responsivity to food cues within the regions of the brain associated with hedonic rewards leads to overconsumption of that food (Berridge et al., 2010). Compared to adolescents who gained weight, whose striatal responsivity increases and leads to future overeating, adolescents who lost weight or maintained body fat show reduced activation of the striatal, insular, and Rolandic opercula in response to palatable foods. Interestingly, the activity in the same neural regions in response to monetary rewards was not increased, suggesting that the hyper-responsivity was specific to food.

The results showed no relationship between neural activation and changes in the pleasure, desire, and reinforcement participants reported in response to a milkshake. However, in adolescents who gained body fat the desire for the milkshake significantly increased from the baseline to the follow up. Taken together, these results may suggest that multiple mechanisms are involved, such as basic motivational and emotional responses as well as cognitive and inhibitory control.

Although increased activity in the striatal regions is mediated by monetary reward among adults (Stice et al., 2011), the absence of modulation in control tasks among adolescents may reflect different structural maturation and neurocognitive strategies between these two age groups. Furthermore, reduction in the amount of time participants worked to earn snack foods or money following the application of a progressive reinforcement paradigm suggests that multiple mechanisms related to social desirability (Leehr et al., 2016), or cognitive strategies may be engaged during the task. As suggested by a novel experimental study (Kemps et al., 2016), restrained eaters who expect to eat high-calorie foods may be able to activate their dieting goal, thereby limiting their food intake or, in this case, food gain. Habitual eating behaviors should therefore be taken into account in future studies, particularly those involving adolescents.

Stice and Yokum's findings shed new light on a rapidly growing area of neuroscience that highlights the neural mechanisms involved in the multifactor etiological process of obesity. These outcomes may have implications for weight loss interventions and the prevention or promotion of healthy diets, habits, and lifestyles since neural hyper-responsivity to food might be a key factor in vulnerability to future weight gain. Encouraging studies suggest that sustained modification of attentional bias may lead to reduced consumption of high-calorie foods, such as chocolate (Kemps et al., 2015), and promote healthy eating (Kakoschke et al., 2014). This may, in turn, reduce the risk of future overeating and weight gain. Identifying the reasons for hyper-responsivity to food cues in people who are gaining weight, are obese, or are candidates for bariatric surgery may be valuable when developing personalized programs in which attention to food cues is addressed.

However, one must use caution when drawing direct links between altered brain responses and obesity since other mediators, such as impulsivity or self-control strategies (Ziauddeen et al., 2015), may be involved, particularly in adolescents. Several developmental neuroimaging studies suggest that, compared to adults and children, adolescents exhibit different responsivity in different neural regions involved in reward processing, such as the ventral striatum and orbitofrontal cortex (Galvan et al., 2006; Gladwin et al., 2011). To generalize these results, as previously noted by Stice and Yokum (2016b), future studies should examine the responsivity of reward-related regions of the brain to a broader range of palatable food, such as salty food and junk food. More work that investigates adolescents is necessary since they are more responsive to reward-related visual food stimuli, such as food advertising (Jastreboff et al., 2014).

## Author contributions

All authors listed, have made substantial, direct and intellectual contribution to the work, and approved it for publication.

### Conflict of interest statement

The authors declare that the research was conducted in the absence of any commercial or financial relationships that could be construed as a potential conflict of interest.
